# Impact of Adherence to a Plant-Based Residential Lifestyle Medicine Program on Cardiometabolic Disease Risk Factors

**DOI:** 10.3390/nu18111683

**Published:** 2026-05-25

**Authors:** Aysha Inankur, Daniel O’Hare, Esteban Arevalo, Ruben Dursus-Élisée, Lyndetta P. Schwartz, Samara R. Sterling

**Affiliations:** 1 Wildwood Lifestyle Center, Wildwood, GA 30757, USA; rdursuselisee@gmail.com; 2 Department of Earth and Biological Sciences, Loma Linda University, Loma Linda, CA 92354, USA; dohare@students.llu.edu; 3 Division of Cardiology, Department of Medicine, Loma Linda University Health, Loma Linda, CA 92354, USA; estebanarevalo.md@gmail.com; 4 Kettering Medical Center, Kettering, OH 45429, USA; lyndi.schwartz@ketteringhealth.org; 5 The Peanut Institute, Albany, GA 31707, USA; samara.sterling@gmail.com; 6 Department of Public Health, Andrews University, Berrien Springs, MI 49104, USA

**Keywords:** Mediterranean diet, MEPA III, vegan, whole-foods, plant-based, cardiometabolic disease, meat intake, water intake, weight loss, residential lifestyle medicine

## Abstract

**Background:** Residential lifestyle medicine programs have documented immediate and long-term improvements in cardiometabolic risk factors. Despite this, adherence among participants varies in such programs, limiting the positive outcomes that can be achieved. This study aimed to assess how adherence to positive lifestyle behaviors correlates with cardiometabolic risk factors at the end of a residential lifestyle medicine program and at three or more months of follow-up. **Methods:** Patients enrolled in a NEWSTART^®^ lifestyle medicine program were invited to participate in this prospective chart review. Outcomes included changes in BMI, blood pressure, medication and supplement use, cardiometabolic disease biomarkers, Mediterranean eating pattern, meat intake, and other lifestyle behaviors. **Results:** Among 109 adults (78% female; 62% overweight or obese) enrolled in a 6- to 39-day (mean 14.5-day) residential intervention, meat intake reduced by 3.2 servings/week, MEPA III scores increased by 2.3, water intake increased by 2.1 glasses/day, and exercise increased by 193 min/week (all *p* < 0.01). From baseline to end of program, reductions were noted in blood glucose (−5.3 mg/dL, *p* = 0.01), total cholesterol (−16.0 mg/dL, *p* < 0.01), LDL cholesterol (−11.0 mg/dL, *p* < 0.01), HDL cholesterol (−2.0 mg/dL, *p* < 0.01), triglycerides (−13 mg/dL, *p* < 0.01), serum creatinine (−0.03 mg/dL, *p* = 0.049), systolic blood pressure (−6.0 mmHg, *p* < 0.01), diastolic blood pressure (−3.0 mmHg, *p* = 0.01), and weight (−3.2 kg, *p* < 0.01). At a mean of 8.6 months follow-up, reductions in triglycerides (14.9 mg/dL, *p* = 0.03) and weight (2.8 kg, *p* < 0.01) from baseline were sustained, and water intake increased 20% from baseline (1.1 glasses/day, *p* = 0.01). Improved adherence to a Mediterranean eating pattern score, increase in water intake and reductions in meat intake and BMI predicted favorable health outcomes. **Conclusions:** Participation in the lifestyle medicine program was associated with improvements in cardiometabolic risk factors during intervention and at follow-up. These outcomes correlated with adherence to positive lifestyle behaviors. Sustained weight reduction as well as dietary and cardiometabolic improvements in our participants suggest the NEWSTART^®^ intervention may hold promise for maintaining cardiometabolic health.

## 1. Introduction

According to the World Health Organization, chronic or noncommunicable diseases (NCDs) account for 75% of non-pandemic-related deaths globally [1]. Chronic disease burden in the US largely stems from a short list of risk factors—tobacco use, unhealthy diet, physical inactivity, alcohol consumption, high blood pressure, and hyperlipidemia [2]. Lifestyle interventions to reduce these risk factors are key to preventing and treating chronic disease [2].

Residential intervention programs allow participants to fully immerse themselves in healthy lifestyle behaviors by providing meals, coaching, and exercise training. While variations in approaches may exist across interventions, many utilize similar principles in their application. For example, Weimar Institute of Health and Education applied the NEWSTART^®^ approach in their program (NEWSTART^®^ stands for Nutrition, Exercise, Water, Sunshine, Temperance, Air, Rest, and Trust in divine power.) [3]. The results of this 18-day lifestyle intervention include reductions in body weight, total and LDL-cholesterol, fasting blood glucose, and blood pressure [4,5,6,7,8]. A one-month residential lifestyle intervention involving a fiber-rich, low-fat diet and exercise included such long-term benefits as cardio-metabolic risk factor reduction at one to five years of follow-up [9]. Benefits at follow-up correlated with maintenance of the new lifestyle [9].

To date, published follow-up outcomes for programs using the NEWSTART^®^ approach have been limited. Symptoms of peripheral diabetic neuropathy were reduced at 1–4 years of follow-up in 16 of 17 NEWSTART^®^ program participants [10]. At follow-up, these persons reported following 71% of the diet and exercise recommendations [10]. Immediate outcomes of residential programs, however, have not been linked with adherence to lifestyle behaviors or length of residential program. Consequently, this study aimed to demonstrate the impact of a residential lifestyle medicine program, using NEWSTART^®^, on cardiometabolic disease risk factors at the end of the program and at three or more months of follow-up. We investigated potential relationships between changes in lifestyle factors over the course of the program and improvements in cardiometabolic risk factors. Outcomes were correlated with adherence to NEWSTART^®^ lifestyle behaviors and time spent in the residential program.

## 2. Materials and Methods

### 2.1. Study Design

The study was a prospective chart review of patients enrolled in the residential program at Wildwood Lifestyle Center. Residential patients who arrived during a 6-month period from November 2021 through May 2022 were invited to participate. Eligible patients were non-pregnant, non-incarcerated adults, at least 18 years of age, and were able to read and speak English. After receiving written informed consent, electronic health records (EHR) were reviewed by the principal investigator. Participants were excluded from analysis if they had been diagnosed with dementia.

At follow-up, two attempts to schedule a phone interview were made via text messages from a study-specific phone number. If the participant could not be reached by text, an email was sent with a link to an electronic survey. If the participant did not respond to the text or email, a paper questionnaire was mailed to the participant with a pre-addressed and stamped return envelope.

### 2.2. Residential Intervention Program

The residential program was typically an 11- to 25-day lifestyle intervention. There were three versions of the intervention: medical, mental health, and life alignment. The medical program included three visits with a licensed medical provider (a medical doctor or nurse practitioner), an optional stress assessment by a licensed clinical social worker (LCSW), and two visits with a nutritionist. The mental health program included a single assessment by a medical provider, four counseling sessions with a LCSW, and two visits with a nutritionist. Nutrition consultations were scheduled at the start and end of the residential phase. The life alignment program included a brief assessment by a medical provider and no individual visits with a LCSW or nutritionist. Group classes included informative sessions on the metabolic syndrome, healthy eating, exercise, and stress management, as well as hands-on cooking classes. The schedule of the residential program is outlined in Table A1.

#### 2.2.1. Nutrition

The diet consisted of whole plant foods served in two or three meals daily. When eating two meals daily, participants skipped the evening meal. Meals were served buffet style. All participants received group education on meal planning using a vegan version of the MyPlate method [11,12]. Recommended portions varied by participant. Consumption of alcohol and nicotine was not allowed (Table 1).

#### 2.2.2. Physical Activity

All participants were encouraged to exercise for 20 min after each meal and to walk 10,000 steps/day, barring medical contraindications. Steps were measured by a pedometer. Daily step counts were reported to lifestyle educators. Participants who were unable to walk were encouraged to use a stationary bicycle or elliptical machine.

#### 2.2.3. Spiritual Support

Participants were invited to join a group for Bible discussion and prayer that met daily. Participants could individually meet with a chaplain during their stay at Wildwood Lifestyle Center. Participants were invited to attend worship services on Friday evenings and Saturday mornings in a nearby church. Religious activities and chaplain visits were optional.

#### 2.2.4. Hydrothermal Therapy and Manual Treatments

Participants received hydrothermal therapy treatments on weekdays and two manual treatments weekly. Hydrothermal therapy treatments consisted of passive hot- and cold-water therapy and included contrast baths, sitz baths, dry sauna, steam baths, and hot-to-cold showers [13,14,15,16,17]. Manual therapies were similar to Swedish massage [18]. While passive heat therapy has known systemic anti-inflammatory effects; specific hydrothermal and manual treatments were chosen based on the medical concerns of individual participants [19].

### 2.3. Follow-Up Program

For 12 weeks following the residential program, participants were offered telephone interviews and bi-weekly classes. Classes were conducted via Zoom. Persons who were unable to use Zoom received weekly phone calls. Class time included five minutes for welcome, religious reflection, and guest introduction; 35–40 min for the topic of discussion; and 15 min for questions, answers, goal- and reminder-setting, and prayer. The webinar titles were as follows: Meal Prep Tips; Questions and Answers with Lifestyle Coach; Establishing a Schedule; Ensuring Success; The Forgotten Remedy—Exercise; How to Fight Inflammation; Questions and Answers on Hydrothermal Therapy; Celebrating Testimonies and Experiences; Brain Health; Immune System; Cancer; and Stress Management.

### 2.4. Variables and Outcome Measures

Differences in data collection during the medical, mental health, and life alignment programs are depicted in Table 2. The time of data collection is indicated by the number of days into the program, with day 1 being the day of arrival.

#### 2.4.1. Anthropometric Measures

Height was measured with shoes removed using a wall-mounted stadiometer at the initial medical visit. Height was self-reported if the participant was unable to stand. Weight was measured using a calibrated digital medical scale, AmCells WWS-500 Digital Doctors Scale (Am-Cells Corp., Vista, CA, USA). Participants removed outer clothing. Weight was not adjusted for shoes but was recorded as it appeared on the scale. Weight was recorded in pounds. During follow-up, weight was self-reported. Waist circumference was measured just proximal to anterior superior iliac crests [20].

#### 2.4.2. Blood Pressure

Automated blood pressure assessments were obtained using the Omron Model Bp710n (Omron, Lake Forest, IL, USA) with regular cuff. The upper arm circumference was assessed at the initial medical visit, and a manual sphygmomanometer with a large cuff was used as needed. During the follow-up phase, blood pressure was self-reported.

#### 2.4.3. Biochemical Measures

Fasting blood samples were collected to assess the serum concentrations of total cholesterol (TC), triglycerides (TG), low-density lipoprotein cholesterol (LDL), high-density lipoprotein cholesterol (HDL), blood glucose (BG), high-sensitivity C-reactive protein (HS-CRP), creatinine and insulin. Insulin resistance was estimated by calculating the homeostasis model assessment-insulin resistance (HOMA-IR) index [21,22]. The glomerular filtration rate (eGFR) was calculated by the CKD-EPI Equation with creatinine [23].

At T1, blood was drawn at Wildwood Lifestyle Center and analytes were measured by Quest Diagnostics(Quest Diagnostics, Tucker, GA, USA) using a Beckman Coulter Automated Chemistry Analyzer; AU480/680 and 5800 series (Beckman Coulter, Inc., Brea, CA, USA). At T2, blood was drawn at Wildwood Lifestyle Center and analyzed at Quest Diagnostics or similar outpatient lab using Siemens Vista 1500 (Siemens Medical Solutions, Malvern, PA, USA) or Seimens Atellica CH (Siemens Medical Solutions, Malvern, PA, USA).

During the follow-up phase, blood was drawn through Quest Diagnostics near the participants’ homes or at similar facilities using Abbott Alinity or Roche Cobas 702 (Roche Diagnostics, Indianapolis, IN, USA).

#### 2.4.4. Survey Instruments

Prior to starting the residential intervention, participants completed a standard history form, from which their age, gender, ethnicity, marital status, religious affiliation, diet type (vegan, vegetarian, omnivore), and medical history were obtained. During the study, participants were also asked about their household income and education level.

All participants were asked to answer a 78-item questionnaire at T1 and T3. Participants in the medical program were also asked to complete this survey at the end of the residential phase (T2). At T1 and T2, survey data was collected via self-administered paper questionnaires followed by in-person interviews to ensure data was complete; at T3, survey data was collected via telephone interviews, online through Foxit eSign or Zoho Sign, or by paper surveys. The follow-up assessment had originally been planned to occur three months after the residential program but was delayed, in many cases, due to difficulty contacting participants.

- Lifestyle measures

Nutrition was assessed by the Mediterranean Eating Pattern for Americans III Diet 23-Item Questionnaire (MEPA III) [24,25]. We used the MEPA III screener because it included components of the whole-food, vegan diet provided to our participants and because of its validity and feasibility in an older population of adults [25]. Total MEPA III scores have been concordant with the VioScreen^TM^ semi-quantitative, 156-question food frequency questionnaire (r = 0.50, *p* < 0.001) [25,26]. Exercise was assessed with the Physical Activity Vital Sign [27]. A non-standardized question was used to assess water intake measured in eight oz. glasses per day.

- Medications and supplements

Current medications and supplements were documented at each medical visit. The average number of anti-hypertensive, anti-hyperglycemic, lipid-lowering and weight-loss medications was compared at T1, T2 and T3. Use of anti-hypertensive, anti-hyperglycemic and lipid-lowering supplements was also assessed and compared at these time points. Use of weight-loss supplements was not assessed, given the limited evidence of efficacy of supplements in reducing body weight [28].

### 2.5. Data Analysis

A pre–post design was used with post-measures at end-of-program and follow-up, without controls. Data were extracted from the electronic health record via manual entry into Microsoft Excel. Within-subject changes between time points (T1 and T2 or T1 and T3) were evaluated using paired-samples *t*-tests. Associations between interventions and physical health metrics were analyzed using multiple linear regression models, with BMI included as a covariate. For analyses between creatinine and water intake, BMI and exercise were included as covariates. Statistical analyses were conducted using IBM SPSS Statistics for Windows, Version 29.0 (IBM Corp., Armonk, NY, USA). The following analyses were performed using Microsoft^®^ Excel for Mac Version 16.78.3 (Microsoft Corp., Redmond, WA, USA): the standard deviation for demographic data was calculated with the function STDEV.P; the standard deviation for weight change was calculated with STDEV.S; changes in body weight and water intake were analyzed with the paired two-sample for means *t*-test; and multiple linear regression involving water intake was performed with the regression function. Statistical significance was set at *p* < 0.05.

## 3. Results

### 3.1. Changes in Physical Health and Lifestyle Choices

**Figure 1:**
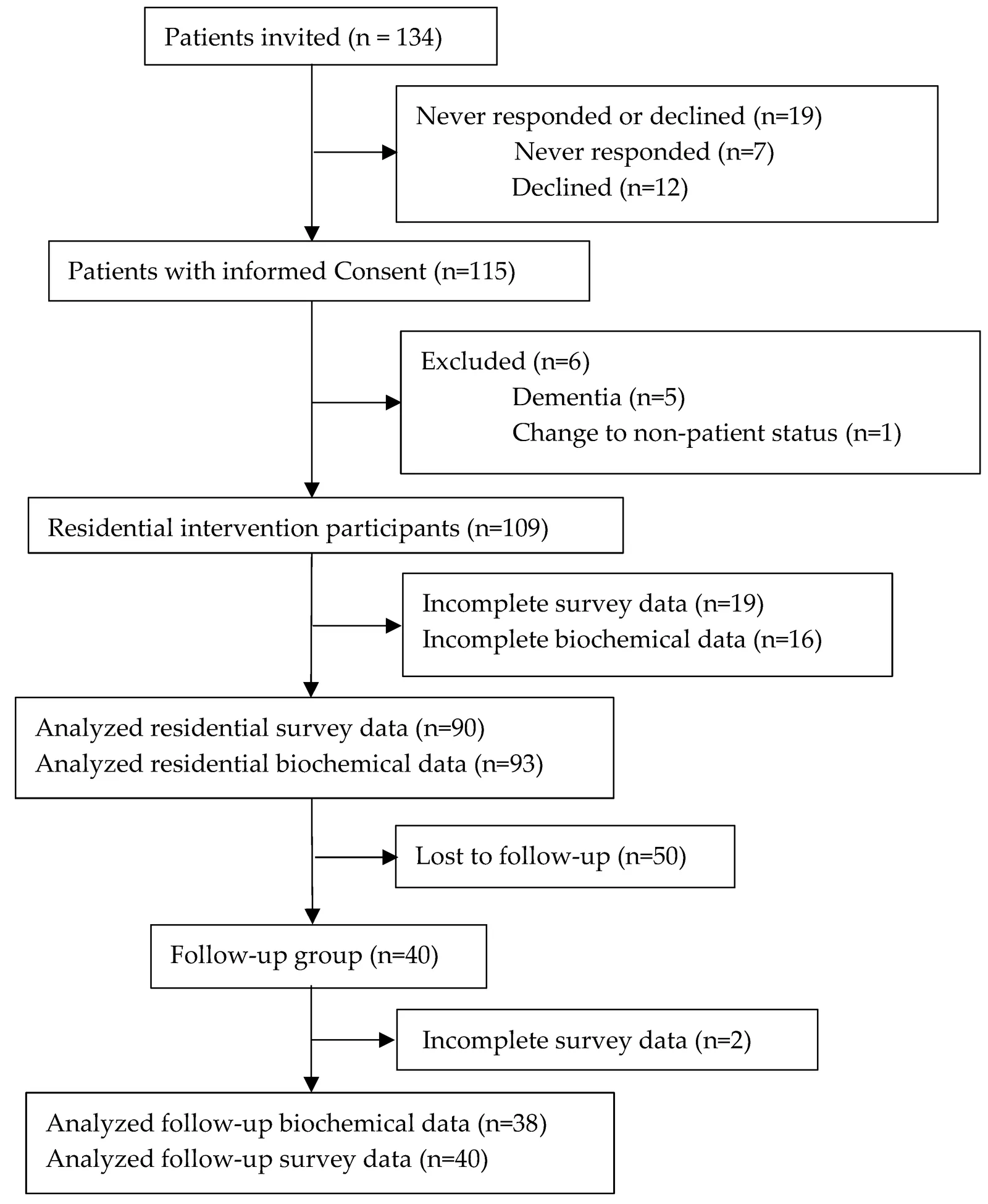
Flow chart of participants through the study.

One hundred and nine adults underwent a 6- to 39-day (mean 14.5-day) residential lifestyle intervention program (Figure 1). Follow-up data was obtained at a mean of 8.6 months (range of 85–575 days) from baseline. We report the cardiometabolic outcomes from the study.

Analyses of changes during the program and at follow-up were calculated from participants with complete data sets. Data from all consented participants, however, were used in cross-sectional analyses (Section 3.4). Table 3 lists the baseline characteristics of consented participants. Table A2 compares the baseline characteristics of T3 survey completers and T3 survey non-completers.

Table 4 and Table 5 compare metrics between baseline and post-intervention, and Table 6 and Table 7 compare these metrics between baseline survey and follow-up survey.

Table 6 and Table 7 display changes in selected metrics from baseline to the time when a follow-up survey was completed after the end of the program.

There was an overall significant improvement in MEPA III score and water intake over the course of the program (Table 4). Meat intake decreased significantly from baseline to the end of the program. The MEPA III score and meat intake did not change significantly among participants who filled out the follow-up survey, although the number of participants who completed this survey (<40) was much smaller than the number of participants (109) who completed the program (Table 7).

#### Anti-Hypertensive, Antihyperlipidemic, Anti-Hyperglycemic and Weight-Loss Medications and Supplements

Over the course of the program, for participants with available data (*n* = 97), 5.2% (*n* = 5) increased anti-hypertensive medication, 17.5% (*n* = 17) reduced or discontinued anti-hypertensive medication, and 41% (*n* = 40) added an anti-hypertensive supplement. During the program, zero participants increased antihyperlipidemic medication, 4.1% (*n* = 4) discontinued, and 1% (*n* = 1) reduced antihyperlipidemic medication (statins), and 4.1% (*n* = 4) of participants added an antihyperlipidemic supplement (Cholesterol Control Complex by Health Thru Nutrition). At baseline, 21% (*n* = 21) of participants were taking anti-hyperglycemic medication and 4.1% (*n* = 4) were taking an anti-hyperglycemic supplement. During the program, 2.1% (*n* = 2) of participants increased their dose of anti-hyperglycemic medication, 6.2% (*n* = 6) of participants discontinued their anti-hyperglycemic medication, 2.1% (*n* = 2) increased anti-hyperglycemic supplementation, and 4.1% (*n* = 4) decreased anti-hyperglycemic supplementation. The number of insulin prescriptions decreased from baseline (*n* = 9) to the end of the program (*n* = 8). During the residential program, one participant took topiramate at a stable dose and zero participants took GLP-1 receptor antagonists. One participant started a GLP-1 receptor antagonist (semaglutide 0.5 mg weekly) during the follow-up period. No participants reported taking liraglutide, tirzepatide, phentermine or orlistat at baseline, end of program, or follow-up.

### 3.2. Amount of Time Spent in the Program

We ran simple linear regression analyses to explore associations between the amount of time participants spent in the program (Program Duration) and positive changes in lifestyle and physical health. We used Program Duration as the predictor variable and tested several health variables, each as a single dependent variable. Table 8 displays the results of the simple linear regression analyses.

Time in the program predicted a decrease in red meat intake. Extended time in a residential program also tended to be associated with greater reductions in HS-CRP, but this association did not reach statistical significance (β = −0.20, *p* = 0.054).

### 3.3. Changes over the Course of the Program

In this section, we examine potential associations between lifestyle changes (predictors) and changes in health outcomes (dependent variables) over the course of the program using pre–post differences in participant data from our health program. We ran multiple linear regression analyses on the survey data to explore relationships between lifestyle changes and improvements in physical health over the course of the program. We assigned the change in lifestyle variables (Program Duration, MEPA III change, meat intake change, exercise change, and water intake change) as the independent variables for each multiple regression analysis, controlling for BMI change. Each analysis included a physical health metric as the dependent variable, and we tested each dependent variable to find all significant multiple regression models.

The following dependent variables were significantly predicted by our chosen independent variables in the multiple regression models: triglycerides change, glucose change, creatinine change, and BMI change.

Within the multiple regression model with triglycerides change as the dependent variable (*p* = 0.045), and controlling for BMI change, only changes in MEPA III scores significantly predicted changes in triglycerides (partial correlation coefficient of −0.305) (Table 9). We also controlled for change in fish intake and change in sugar intake (from non-whole-fruit sources).

In the multiple regression model using change in glucose as the dependent variable and controlling for change in BMI (*p* < 0.01), change in meat intake (partial coefficient of −0.328), exercise change (partial coefficient of 0.230), and BMI change (partial coefficient of 0.611) emerged as significant predictors (Table 9).

In the multiple regression model using change in creatinine levels as the dependent variable and controlling for change in BMI (*p* < 0.01), only a change in exercise significantly predicted a change in creatinine levels (partial coefficient of 0.272) during the program (Table 9). BMI change was significantly positively correlated with creatinine level. The adjusted R^2^ for the model was 0.412. In the multiple regression model using a change in creatinine level as the dependent variable and controlling for changes in BMI and exercise, water intake yielded significant results only from baseline to follow-up. With a *p*-value of 0.04 for the model, water intake negatively correlated with creatinine level (partial coefficient of −0.042; *p* = 0.03). The adjusted R^2^ was 0.119.

In the multiple regression model using a change in BMI as the dependent variable (*p* = 0.01), only change in MEPA III score significantly predicted change in BMI (partial coefficient of −0.274) (Table 9). Adjusted R^2^ was 0.112. In regression models including an interaction between baseline MEPA III and change in MEPA III, the main inverse effect of MEPA-III change on BMI remained (B = −1.03, *p* < 0.01). There was a positive correlation between the interaction terms (B = 0.08, *p* <0.01), suggesting that this association may be stronger among those with lower baseline MEPA III scores.

In the multiple regression models using change in total cholesterol, HDL cholesterol, non-HDL cholesterol, and LDL cholesterol as dependent variables and controlling for change in BMI (*p* < 0.001), none of the independent variables were significant (Table 9). However, BMI change significantly predicted a decrease in all types of cholesterol.

### 3.4. Associations Between Lifestyle and Physical Health: Simple and Multiple Regression

In this section, we pool all observations from the baseline (T1) and end-of-program (T2) surveys to examine overall cross-sectional associations between lifestyle habits (predictors) and health outcomes (dependent variables), independent of change over time. To address missing data in some of the variables for some of the participants, we chose to exclude cases pairwise in the regression analyses.

We ran multiple regression analyses to test several health variables, selecting the following lifestyle choices as independent (predictor) variables: MEPA III score, bedtime, time spent outside, and exercise, while controlling for BMI. Table 10 shows the predictor variables which exhibited statistically significant or nearly significant associations with the studied health benefits.

BMI was the only predictor variable that was able to significantly predict at least one health metric. BMI predicted poor levels of HDL cholesterol, non-HDL cholesterol, triglycerides, c-reactive protein, insulin, and HOMA-IR (Table 10).

## 4. Discussion

We observed associations between participation in a residential lifestyle medicine program and improvements in several cardiometabolic risk factors during the intervention and at follow-up, with increases in Mediterranean eating pattern and water intake and reductions in meat intake and BMI predicting favorable outcomes. The amount of time spent in the program showed a trend toward lower HS-CRP with longer participation (*p* = 0.054), although this latter association did not reach statistical significance (Table 8).

Our results align with prior studies using whole-foods, plant-based diets and Mediterranean eating patterns. The MEPA III screener used to assess adherence to a Mediterranean eating pattern has a score range of 0–21. At T1, the mean score among our participants was 12.2 (range 5–19); at T2, mean was 14.5 (range 9–18); and at T3, the mean was 14.4 (range 7–18). Our scores at baseline appear higher than other reports from populations in the US, showing average MEPA III scores of 8.6 and 10.7 [25,29]. Scores of around 11 and below are thought to reflect low accordance with the Mediterranean diet and to be more in line with a Western diet pattern [24]. In an NHANES population of 2598 older participants, MEPA III scores of ≥10 correlated with a lower risk of stroke and better cognitive performance than scores of <8 [29]. Use of soy isoflavones and soy products has been associated with reduced risk of CVD risk factors in post-menopausal women, and a meta-analysis found an association between soy intake and reduced CVD and CHD risk, particularly in western populations [30,31]. The presence of soy foods in our residential intervention may have mediated some of the metabolic outcomes observed in our mostly post-menopausal female participants. We did not assess the gut microbiota of our participants. Yet, dietary transitions are known to impact the microbiome with major shifts in microbial strains observed as early as 1 day into an intervention [32,33]. Cardiovascular benefits of vegetarian diets may be mediated by an associated increase in the *Prevotella* enterotype [34]. In contrast, diets high in animal protein are known to promote the *Bacteriodes* enterotype associated with CVD [35]. Diet-induced changes in gut microbiota likely influenced our metabolic outcomes. The reduction in BMI seen with our plant-based diet intervention aligns with meta-analyses of RCTs showing weight loss with vegetarian diet interventions without energy restriction [36]. We saw a positive association between weight loss and an increase in Mediterranean eating pattern score. This is not surprising, as meta-analyses have shown Mediterranean diet interventions to result in weight loss in overweight and obese individuals [37,38]. The average weight loss of 3.87% (3.2 kg) during the mean 14.5 days of our residential intervention was not less than the 2.7% (2.2 kg) reported weight loss over 18 days of a similar residential program or the 1.4 kg mean weight loss over seven days of a residential low-fat, vegan intervention [4,39]. Weight lost during the program was maintained in the subset of participants with follow-up data. None of our participants initiated medication for weight loss during the residential phase, and one participant started a relatively low-dose GLP-1 receptor agonist during follow-up, suggesting that weight loss was achieved and maintained by the lifestyle medicine strategies of our program. With obesity affecting 40.3% of US adults and one in eight adults globally, finding safe, affordable and sustainable weight-loss methods is crucial [40,41]. The NEWSTART^®^ lifestyle intervention may represent one such method.

Whole-food, plant-based diet interventions of one to eight weeks have been associated with reductions in total and LDL cholesterol [39,42]. The reduction in HDL-cholesterol in our study population was consistent with other reports on vegan diet interventions [39,43]. Prediction of fasting blood glucose reduction with reduction in meat intake is in line with findings of the seven-day low-fat vegan diet on fasting blood glucose and a 24-week low-fat vegan diet intervention showing reduced insulin resistance [39,44]. Participants in our residential program were provided vegan meals; thus, meat intake would be expected to decrease by 100% rather than 96.6%. The small reported meat intake by end of program was likely due to participants reporting intake over a one-month period, extending back prior to the program. The reduction in triglycerides with a positive change in Mediterranean pattern in our study aligns with a meta-analysis of Mediterranean diet interventions [45].

We report several novel findings related to the residential NEWSTART^®^ intervention. Our study is the first to associate a significant increase in MEPA III scores, water intake, minutes of weekly exercise and a significant reduction in serum creatinine with this residential intervention. We found that, relative to baseline, triglycerides and body weight were significantly reduced and water intake was significantly increased at follow-up. We also noted that improved adherence to a Mediterranean eating pattern score, increase in water intake and reductions in meat intake and BMI during the residential program predicted favorable metabolic outcomes. Metabolic health improvements had previously been demonstrated after a five-day intervention involving seven of the NEWSTART^®^ behaviors, with apparent exclusion of a religious component. Weight, BMI, blood pressure, total cholesterol, HDL-C, LDL-C, and fasting serum glucose decreased for Karpalo health-immersion program participants at their 6-week follow-up [46]. In contrast, our program involved all eight aspects of NEWSTART^®^ and had a longer follow-up interval.

We recommended a general goal of at least 10,000 steps/day due to mortality benefit with this dose of exercise in people with pre-diabetes and diabetes in a general population under 60 years of age [47,48]. We specifically encouraged participants to walk after meals. In persons with type 2 diabetes, 15 to 30 min of aerobic or resistance exercises initiated 30 min after a meal has been shown to significantly reduce post-meal glucose peaks [49]. Glucose peaks are expected to occur 30–60 min after eating in healthy individuals and 60–120 min after eating in people with diabetes [50,51,52]. Considering walking as a feasible form of aerobic activity for most participants, we recommended a 20 min walk immediately after meals to blunt post-prandial glucose peaks. Physical activity was monitored by pedometers, which participants wore daily. While clinical charts contained daily step counts, this data was not extracted for analysis. We, instead, analyzed self-reported exercise time (Physical Activity Vital Sign) as this data could be collected at T1, T2 and T3 [27]. Surprisingly, our data showed a short-term association between increased exercise and increased fasting glucose (Table 9). Exercise programs carried out over one to 12 weeks have been shown to increase insulin sensitivity, likely due to improved mitochondrial function [53,54]. Thus, we expected change in exercise to negatively correlate with change in fasting blood glucose. The positive association between an increase in exercise and in fasting glucose in our data may be transient and may reflect other confounding factors among those whose exercise habits changed in our program. The apparent correlation between increased serum creatinine after short-term increases in moderate physical activity may be due to either transient rises in creatinine during exercise or the short-term duration of the association. Strenuous and prolonged physical activity has been associated with increased serum creatinine [55]. Conversely, moderate exercise, defined as two to six times per week, has been linked with reduced decline in kidney function over several years [56]. The positive association we found between change in exercise and change in creatinine may be due to an acute increase in exercise over the short duration of our study. Exercise can cause a transient rise in creatinine levels even when physiology remains unchanged [57]. Additionally, the positive effects of moderate exercise on kidney function may not emerge until weeks to months have passed [58].

Coaching to increase water intake has been associated with improved kidney function over a twelve-month period [59]. In our study, participants were generally encouraged to consume at least 8 cups of water per day. At follow-up, creatinine decreased from baseline with increased water intake.

Lifestyle program participants, on average, experienced weight loss and reduced BMI over the course of the program and from baseline to follow-up (Table 5 and Table 7). Weight loss mediated multiple benefits seen in the program. Reduction in BMI predicted reductions in blood glucose and in cholesterol (Table 9). These glycemic and non-HDL cholesterol results are consistent with prior reports [60,61,62]. As was the case in our study, weight loss on a normal-protein diet has correlated with a reduction in serum creatinine [63].

A strength of this study was all T1 blood samples being analyzed by Quest Diagnostics, with most T2 and T3 blood samples analyzed by Quest Diagnostics. Blood for lipid and metabolic panels at T2 and T3 were analyzed by one and three additional labs, respectively. Blood was consistently analyzed using standard laboratory techniques. Four of the five laboratories used equipment certified by the Cholesterol Reference Method Laboratory Network, recommended by the CDC [64,65]. The one lab, LabCorp, for which we were unable to identify make or model of lipid analyzer has demonstrated consistent lipid results compared with Quest Diagnostics [66]. At T2 HS-CRP was measured via the Siemens Dimension Vista 1500 and the Roche c503 system used by Quest Diagnostics. Data from these two HS-CRP assay systems, however, are known to be comparable and correlated [67]. At T2, glucose and creatinine were measured by the Siemens Dimension Vista 1500 in addition to the Beckman AU5800 used by Quest Diagnostics. While studies directly comparing creatinine results using these two systems have not been found, both creatinine tests systems are traceable to the international reference standard, the Isotope Dilution Mass Spectrometry (IDMS) [68,69]. Glucose measured by the Beckman AU5800 and Siemens Advia assays showed excellent peer performance attributes; and glucose measured by the Siemens Advia has correlated with results from the Siemens Dimension Vista 1500 (Siemens Medical Solutions, Malvern, PA, USA), used in our study, with Pearson correlation coefficients (r) ≥ 0.96 [70].

Our study was limited by inconsistencies in data collection. Blood pressure was typically measured once at clinic appointments. The recommended standard is for multiple blood pressure readings to be averaged at each assessment [71]. Our deviation from this standard may have caused inaccuracy. At T2, insulin levels were measured via the Roche Elecsys electrochemiluminescence immunoassay and the Siemens Atellica chemiluminescent immunoassay, used by Quest Diagnostics [72,73]. While both methods claim traceability to the WHO 66/304 standard, inter-assay variability exists between these two immunoassays and may have affected insulin results for these two individuals [74]. Nine participants saw a medical provider whose last clinic visit occurred one day early, causing biochemical tests, weight, and blood pressure to be measured a day early at T2. This may have reduced the measured impact of the residential intervention in these participants. While surveys were by default self-administered, some were interview-administered. The delivery mode for self-administered surveys was mostly paper, but electronic surveys were also used. As survey questionnaire responses are a product of the interaction between the questionnaire, the respondent and the delivery mode, variation in our delivery modes may have introduced survey bias [75]. Follow-up findings should be interpreted with caution given the relatively small sample size (*n* = 40) compared to the initial cohort (*n* = 109); this may introduce selection bias, with those having more favorable results potentially being more likely to provide follow-up data, which may limit the generalizability of sustained effects [76].

Unmeasured variables may, additionally, have impacted results. Participants in the medical, mental health, and life alignment programs received different numbers of visits from medical providers, nutritionist and LCSW. Differences in the number and type of these consultations may have impacted outcomes. All participants received hydrothermal therapy and manual therapy, which may have impacted their metabolic health. Acute or chronic effects associated with hydrothermal therapy include increased nitric oxide bioavailability, reduced glucose and insulin levels, CRP, and systolic and diastolic blood pressure [77,78]. Meta-analyses have shown that massage may have beneficial effects on cardiometabolic outcomes, including cholesterol, triglycerides, and blood pressure, though these findings remain tentative [79,80]. Without a comparison group which did not receive hydrothermal or manual therapy in our study, we are unable to determine how much these treatments influenced our cardiometabolic outcomes. We did not have data on the number of chaplain visits participants received to assess whether this type of spiritual support was associated with metabolic outcomes. While overall anti-hypertensive, anti-hyperglycemic, and antihyperlipidemic medication use decreased during the residential program and anti-hyperglycemic supplement use decreased as well, use of anti-hypertensive supplements increased markedly. These blood pressure-lowering supplements may have impacted blood pressure results. Less than 5% of participants started an antihyperlipidemic supplement during the program, and the impact of these supplements may have been offset by the equal number of participants who discontinued statin therapy. Also, changes in medications and supplements outside the categories listed above may have impacted program outcomes. Our outcome measures were limited to relatively short-term changes in biochemical markers, blood pressure and body weight. The strength of plant-based diets, however, is their impact on human health over the life-span.

Future research with larger sample sizes, longer follow-up interval, and correlation with medication and supplement use is needed to assess the impact of long-term adherence to the NEWSTART^®^ intervention.

## 5. Conclusions

Participation in a residential lifestyle medicine program incorporating NEWSTART^®^ components was associated with improvements in several cardiometabolic risk factors at the end of the program and among participants with available follow-up data. These outcomes were correlated with adherence to assessed lifestyle behaviors. However, given the observational design, absence of a control group, and limited follow-up sample size, these findings should be interpreted with caution. The results are hypothesis-generating and suggest that such interventions may support improvements in cardiometabolic health, but larger, controlled studies with longer follow-up are needed to confirm these associations and assess long-term sustainability. Weight loss maintenance among our mainly female and mostly overweight or obese participants suggests that the NEWSTART^®^ intervention may hold promise as a tool for helping maintain weight loss in such adults.

## Figures and Tables

**Table 1:** Whole-food plant-based diet.

**Allowed foods** Non-starchy vegetables Starchy vegetables Fruits (whole fresh or frozen) Whole grains Legumes (beans, peas, lentils) and Soy Curls^TM^ Plant-based or non-caloric beverages (unsweetened organic soy milk, nut beverages, water, non-caffeinated tea) All culinary herbs and mild spices Seeds rich in omega-3 fats (ground flaxseed, chia seed)
**Excluded foods** All animal products: Meat, poultry, fish, and seafood; eggs; dairy products Refined flours Packaged vegan meat and cheese replacements Alcoholic beverages Caffeinated beverages Sugar-sweetened beverages Strong spices (black pepper, cayenne pepper, cinnamon, nutmeg, and cloves) Non-nutritive sweeteners (with exception of stevia)
**Limited foods (consume sparingly)** High-fat plant foods: raw or dry-roasted nuts; nut butters, seeds (other than above); coconut; avocado Dried fruits Smoothies Refined soy (non-GMO tofu) Juices (100% fruit or vegetable juices) Added sweeteners (honey, maple syrup, blackstrap molasses) Added oils and creams (olive oil, sesame oil, coconut milk, coconut cream, and unrefined coconut oil used sparingly in baking) Stevia not used in cooking but available for optional use in tea

**Table 2:** Data collection timetable.

Phase	Program Type	Survey ^a^	Cardiometabolic Disease Biomarkers	Weight, Blood Pressure	Height
Phase 1 11-day	Medical	Days 0–2, 10	Days 2 ^b^, 9 ^c^	Days 2, 10	Day 2
	Mental Health	Days 0–2	Day 2 ^d^	Day 2	Day 2
	Life Alignment	Days 0–2	Day 2 ^e^	Day 2	Day 2
Phase 1 25-day	Medical	Days 0–2, 24	Days 2 ^b^, 23 ^c^	Days 2, 24	Day 2
	Mental Health	Days 0–2	Day 2 ^d^	Day 2	Day 2
	Life Alignment	Days 0–2	Day 2 ^e^	Day 2	Day 2
Phase 2	Medical	Week 12+	Week 12+ ^f^	Weeks 12+	Not measured
	Mental Health	Week 12+	Not measured	Weeks 12+	Not measured
	Life Alignment	Week 12+	Not measured	Weeks 12+	Not measured

^a^ Seventy-eight-item questionnaire; ^b^ complete metabolic panel (CMP), fasting lipid panel, high-sensitivity C-reactive protein (HS-CRP) and insulin; ^c^ CMP, fasting lipid panel, HS-CRP and insulin; ^d^ CMP and fasting lipid panel; ^e^ CMP; ^f^ CMP and fasting lipid panel.

**Table 3:** Participant characteristics at baseline.

Description	*n* = 109 (Except as Noted)
Program Type, *n* (%)	
Medical	94 (86%)
Mental Health	9 (8%)
Life Alignment	6 (6%)
Age, years (SD)	61.7 (13.5)
Sex, *n* (%)	
Female	85 (78%)
Male	24 (22%)
Marital Status, *n* (%)	
Single	17 (16%)
Married	53 (48%)
Separated	6 (6%)
Divorced	19 (17%)
Widowed	14 (13%)
Ethnicity, *n* (%)	
African American	68 (62%)
White	31 (28%)
Hispanic	7 (6%)
Asian	4 (4%)
Education Attained, *n* (%)	
Less than high school	4 (4%)
High school graduate	17 (15%)
Some post high school	29 (27%)
College graduate or more	27 (25%)
Graduate degree	26 (24%)
Missing data	6 (5%)
Annual Household Income, *n* (%)	
<$10,000	6 (6%)
$10,000–$24,999	6 (6%)
$25,000–$49,999	14 (13%)
$50,000–$74,999	12 (11%)
$75,000 or more	29 (26%)
Declined to answer	10 (9%)
Missing data	32 (29%)
Religious Affiliation	
Seventh-day Adventist	85 (78%)
Catholic	3 (3%)
Christian	14 (13%)
Baptist	2 (2%)
Pentecostal	2 (2%)
Messianic Jew	1 (1%)
Unknown	2 (2%)
Weight status, *n* (%)	
Underweight (BMI < 18.5)	1 (1%)
Normal Weight (BMI 18.5–24.9)	40 (37%)
Overweight (BMI 25–29.9)	25 (23%)
Obese (BMI ≥ 30)	43 (39%)
Weight, kg (SD)	81.3 (28.6)
BMI (SD)	29.3 (8.8)
Diagnoses, *n* (%)	
Pre-diabetes	15 (14%)
Type 2 diabetes	21 (19%)
Coronary artery disease	7 (6%)
Hypertension	67 (61%)
Hyperlipidemia	72 (66%)
History of cancer	25 (23%)
Medication use, *n* (%)	
Statin or ezetimibe	26 (24%)
Anti-hypertensive	56 (51%)
Anti-hyperglycemic	21 (19%)
Dietary pattern, *n* (%)	
Vegan	16 (15%)
Vegetarian	34 (31%)
Omnivore	59 (54%)

**Table 4:** Lifestyle changes from start to end of residential program.

Lifestyle Change	N	Baseline (T1) Mean (SD)	Program End (T2) Mean (SD)	Mean Change (95% CI)	Percent Change	*p*-Value
MEPA III Score	90	12.2 (2.5)	14.5 (1.9)	2.3 (1.6, 2.9)	18.9%	**<0.01**
Meat Intake	90	3.3 servings/week (5.4)	0.1 serving/week (0.7)	−3.2 (−4.4, −2.1)	−96.6%	**<0.01**
Exercise	79	119 min/week (126)	312 min/week (194)	193 (146, 240)	162%	**<0.01**
Water Intake	89	5.5 glasses/day (2.4)	7.6 glasses/day (2.5)	2.1 (1.5, 2.6)	38.2%	**<0.01**

Statistically significant *p* values are in **bold**.

**Table 5:** Changes in laboratory metrics over the course of the program.

Physiological Health Metric	N	Baseline (T1) Mean (SD)	Program End (T2) Mean (SD)	Mean Change (95% CI)	Percent Change	*p*-Value
eGFR (mL/min/1.73 m^2^)	90	81.9 (22.8)	83.2 (21.7)	1.3 (−0.6, 3.1)	1.56%	0.18
Weight (kg)	93	82.0 (29.2)	78.9 (26.8)	−3.2 (−4.4, −2.0)	−3.87%	**<0.01**
BMI (kg/m^2^)	93	29.6 (9.0)	28.5 (8.2)	−1.1 (−1.6, −0.7)	−3.72%	**<0.01**
Creatinine (mg/dL)	91	1.03 (1.1)	1.00 (1.0)	−0.03 (−0.1, 0)	−2.91%	**0.049**
Fasting Glucose (mg/dL)	91	92.9 (24.0)	87.6 (16.1)	−5.3 (−9.2, −1.4)	−5.71%	**0.01**
Total Cholesterol (mg/dL)	90	183 (44.0)	168 (41.4)	−16.0 (−20.2, −10.9)	−8.2%	**<0.01**
LDL Cholesterol (mg/dL)	90	106 (36.6)	95 (34.5)	−11.0 (−15.1, −7.2)	−10.4%	**<0.01**
HDL Cholesterol (mg/dL)	90	56 (16.1)	54 (16.5)	−2.0 (−4.1, −0.9)	−3.57%	**<0.01**
Non-HDL Cholesterol (mg/dL)	90	127 (41)	114 (38)	−13 (−17.3, −8.8)	−10.2%	**<0.01**
Triglycerides (mg/dL)	90	113 (61)	100 (42)	−13 (−20.9, −4.5)	−11.5%	**<0.01**
Insulin (mIU/L)	83	9.7 (7.8)	8.7 (7.8)	−1.0 (−3.0, 1.0)	−10.3%	0.31
HOMA-IR	83	2.38 (2.40)	1.87 (1.58)	−0.5 (−1.0, 0)	−21.4%	0.05
HS-CRP (mg/L)	90	3.34 (3.26)	3.28 (3.41)	−0.1 (−0.6, 0.4)	−1.8%	0.80
Systolic Blood Pressure (mmHg)	93	127 (18)	121 (16)	−6.0 (−8.9, −3.1)	−4.72%	**<0.01**
Diastolic Blood Pressure (mmHg)	93	80 (11)	77 (9)	−3.0 (−4.7, −0.7)	−3.75%	**0.01**

Statistically significant *p* values are in **bold**.

**Table 6:** Lifestyle changes from the start of the program to follow-up.

Lifestyle Change	N	Baseline (T1) Mean (SD)	Follow-Up (T3) Mean (SD)	Mean Change (95% CI)	Percent Change	*p*-Value
MEPA III Score	40	12.4 (2.6)	12.8 (2.6)	0.4 (−0.5, 1.3)	3.2%	0.40
Meat Intake	39	2.1 servings/week (3.8)	1.5 servings/week (2.8)	−0.6 (−1.9, 0.7)	−28.6%	0.37
Exercise	38	128 min/week (100)	150 min/week (103)	22 (−20, 64)	17%	0.24
Water Intake	40	5.5 glasses/day (2.2)	6.4 glasses/day (2.2)	1.1 (0.9, 1.9)	20.0%	**0.01**

Statistically significant *p* values are in **bold**.

**Table 7:** Changes in laboratory metrics from beginning of program to the time of the follow-up survey.

Physiological Metric	N	Baseline (T1) Mean (SD)	Follow-Up (T3) Mean (SD)	Mean Change (95% CI)	Percent Change	*p*-Value
eGFR (mL/min/1.73 m^2^)	37	87.0 (23.8)	86.9 (23.4)	−0.2 (−3.9, 3.6)	−0.1%	0.91
Weight (kg)	36	79.7 (24.5)	76.9 (24.1)	−2.8 (−1.5, −4.2)	−3.5%	**<0.01**
BMI (kg/m^2^)	34	28.6 (7.5)	27.6 (7.3)	−1.0 (−1.6, −0.5)	−3.5%	**<0.01**
Creatinine (mg/dL)	37	0.98	0.98	0.0 (−0.04, 0.05)	0%	0.88
Fasting Glucose (mg/dL)	37	95.3 (26.1)	96.9 (31.9)	1.6 (−6.1, 9.3)	1.7%	0.67
Total Cholesterol (mg/dL)	39	179.8 (47.4)	177.0 (46.9)	−2.8 (−10.7, 5.0)	−1.6%	0.47
LDL Cholesterol (mg/dL)	39	101.8 (40.0)	101.7 (40.8)	−0.1 (−7.3, 7.0)	−0.1%	0.97
HDL Cholesterol (mg/dL)	39	56.8 (17.1)	56.1 (17.1)	−0.7 (−3.2, 1.9)	−1.2%	0.59
Non-HDL Cholesterol (mg/dL)	37	122.2 (41.7)	120.4 (42.7)	−1.8 (−9.5, 5.9)	−1.5%	0.64
Triglycerides (mg/dL)	37	108.6 (56.8)	93.7 (39.9)	−14.9 (−27.9, −1.9)	−13.7%	**0.03**
Systolic Blood Pressure (mmHg)	34	127.0 (13.5)	125.6 (16.2)	−1.4 (−7.5, 4.7)	−1.1%	0.64
Diastolic Blood Pressure (mmHg)	34	78.1 (9.4)	74.6 (8.0)	−3.5 (−7.0, 0.09)	−4.48%	0.06

Statistically significant *p* values are in **bold**.

**Table 8:** Results of outcome variables, using Program Duration as the predictor variable.

Outcome Variable	Relationship with Program Duration	*p*-Value
HS-CRP	Negative	0.05
Meat Intake	Negative	**<0.01**
Exercise	Positive	0.07
MEPA III	None	0.15

Statistically significant *p* values are in **bold**.

**Table 9:** Multiple regression analyses using seven predictor variables.

Tested Dependent Variable	Model Significance	Predictor (Independent) Variables	Partial Coefficients
Triglycerides Change *	*p* = 0.045	MEPA III Change	−0.31
Glucose Change	*p* < 0.01	Meat Intake	−0.33
		Exercise Change	+0.23
		BMI Change	+0.61
Creatinine Change	*p* < 0.01	Exercise Change	+0.27
		BMI Change	+0.59
BMI Change	*p* = 0.01	MEPA III Change	−0.27
Total Chol	*p* < 0.01	None except BMI Change	
HDL Chol	*p* < 0.01	None except BMI Change	
Non-HDL Chol	*p* < 0.01	None except BMI Change	
LDL Chol	*p* < 0.01	None except BMI Change	

* For multiple regression using triglycerides change, we also controlled for fish intake and refined sugar intake.

**Table 10:** Associations between predictor variables and laboratory and vitals data using multiple regression models.

Independent (Predictor) Variable	Model Significance	Dependent Variables	Association
BMI	**0.03**	HDL Cholesterol	Negative
	**0.04**	Non-HDL Cholesterol	Positive
	**<0.01**	Triglycerides	Positive
	**<0.01**	HS-CRP	Positive
	**<0.01**	Insulin	Positive
	**<0.01**	HOMA-IR	Positive
MEPA III Score	0.07	Non-HDL Cholesterol	Negative
Later Bedtime	0.06	HOMA-IR	Positive

Statistically significant *p* values are in **bold**.

## Data Availability

The original contributions presented in this study are included in the article. Further inquiries can be directed to the corresponding author.

## Appendix A

**Table A1:** Schedule for the residential lifestyle intervention *.

**Time**	**Day 1** **Sunday**	**Day 2** **Monday**	**Day 3** **Tuesday**	**Day 4** **Wednesday**		**Day 5** **Thursday**	**Day 6** **Friday**	**Day 7** **Saturday**
6:00	Patients arrive at Wildwood Lifestyle Center	Blood draw	Wake Up					
6:15			Drink Water					
6:30		Worship						
7:00		Breakfast						
8:00		Walk and Personal Devotional Time						
9:00		Visits: medical, nutritionist and LCSW	Health Lecture			Visits: medical, nutritionist and LCSW	Hydrotherapy	Church Services
10:00			Free Time					
10:30		Mental Health Lecture	Mental Health Group Activity		Mental Health Lecture	Mental Health Group Activity		
11:30		Visits: medical, nutritionist and LCSW	Health Lecture/Cooking Class		Cooking School and Nutrition Tips	Cooking School and Nutrition Tips		
12:00							Cooking Class	
13:00		Lunch						
14:00	Registration	Walk						Recreation
15:00		Hydrotherapy	Hydrotherapy		Hydrotherapy	Hydrotherapy	Health Lecture	
16:00							Free Time	
17:00			Creative Expression Class					
18:00	Optional Light Supper							
19:00	Orientation	Charcoal Lecture	Ministry of Healing Group Session			Health Lecture	Worship	Testimony
**Time**	**Day 8** **Sunday**	**Day 9** **Monday**	**Day 10** **Tuesday**		**Day 11** **Wednesday**	**Day 12** **Thursday**	**Day 13** **Friday**	**Day 14** **Saturday**
6:00	Wake Up	Blood draw	Wake Up					
6:15			Drink Water					
6:30	Worship							
7:00	Breakfast							
8:00	Walk and Personal Devotion Time							
9:00	Free Time	Health Lecture	Visits: medical, nutritionist and LCSW		Health Lecture	Free Time	Free Time	Church Services
10:00	Health Lecture	Free Time			Free Time			
10:30		Mental Health Lecture	Mental Health Group Activity		Mental Health Lecture			
11:00	Free Time							
11:30		Cooking School and Nutrition Tips	Visits: medical, nutritionist and LCSW		Cooking School and Nutrition Tips			
12:00								
1:00	Lunch							
2:00	Free Time	Walk						Recreation
3:00	Health Lecture	Hydrotherapy	Hydrotherapy or Health Lecture		Hydrotherapy	Free Time	Free Time	
4:00	Free Time		Hydrotherapy					
5:00	Wildwood Natural Foods Market Field Trip		Hydrotherapy or Creative Expression Class					
6:00	Optional Light Supper							
7:00	Health Lecture/DVD	Health Lecture	Ministry of Healing Group Session		Graduation	Free Time	Worship	Free Time

* In the 11-day program, participants left on day 12. In the 25-day program, participants stayed for days 1–14 followed by a repeat of days 1–11.

**Table A2:** Comparison of baseline characteristics of T3 survey completers and non-completers.

Description	T3 Survey Completers	Non-T3 Survey Completers	*p*-Value
Age (years)	62 (mean)	61 (mean)	0.81
Female, *n* (%)	33 (83%)	52 (75%)	0.39 (chi-squared)
African American, *n* (%)	19 (51%)	38 (58%)	0.54 (chi-squared)
Education > high school, *n* (%)	30 (79%)	52 (80%)	0.90 (chi-squared)
Household income ≥ $50,000, *n* (%)	16 (61%)	39 (76%)	0.17 (chi-squared)
BMI (kg/m^2^) (mean ± SD)	28.8 ± 7.1	29.6 ± 9.8	0.66
Obesity (BMI ≥ 30), *n* (%)	15 (38%)	28 (41%)	0.75 (chi-squared)
Hypertension, *n* (%)	21 (52%)	46 (67%)	0.14 (chi-squared)
Hyperlipidemia, *n* (%)	21 (53%)	53 (77%)	0.01 (chi-squared)
Type 2 diabetes, *n* (%)	8 (20%)	13 (19%)	0.88 (chi-squared)
Statin/ezetimibe use, *n* (%)	8 (20%)	17 (25%)	0.58 (chi-squared)
Anti-hypertensive medication use, *n* (%)	19 (48%)	37 (54%)	0.54 (chi-squared)
Anti-hyperglycemic medication use, *n* (%)	9 (25%)	12 (16%)	0.51 (chi-squared)
HS-CRP, mg/L (mean ± SD)	2.7 (2.8)	3.6 (3.4)	0.19
A1C (%) (mean ± SD)	5.9 (1.1)	6.0 (1.1)	0.63
Total Chol (mg/dL) (mean ± SD)	179 (45)	184 (42)	0.56
MEPA III Score (mean ± SD)	13 (3)	12 (3)	0.11
Exercise (minutes/day*days/week)	137	110	0.30
Meat intake (servings/day) (mean ± SD)	0.11 (0.26)	0.21 (0.47)	0.16
